# Extracellular and Intracellular Lanthanide Accumulation in the Methylotrophic *Beijerinckiaceae* Bacterium RH AL1

**DOI:** 10.1128/AEM.03144-20

**Published:** 2021-06-11

**Authors:** Carl-Eric Wegner, Martin Westermann, Frank Steiniger, Linda Gorniak, Rohit Budhraja, Lorenz Adrian, Kirsten Küsel

**Affiliations:** a Institute of Biodiversity, Aquatic Geomicrobiology, Friedrich Schiller University, Jena, Germany; b Electron Microscopy Center, Jena University Hospital, Jena, Germany; c Helmholtz Centre for Environmental Research, UFZ, Department of Isotope Biogeochemistry, Leipzig, Germany; d Helmholtz Centre for Environmental Research, UFZ, Department of Environmental Biotechnology, Leipzig, Germany; e German Center for Integrative Biodiversity Research (iDiv) Halle-Jena-Leipzig, Leipzig, Germany; University of Tokyo

**Keywords:** *Beijerinckiaceae*, methylotrophy, lanthanides, EDX, freeze fracture electron microscopy, metallomics, RNA-seq

## Abstract

Recent work with Methylorubrum extorquens AM1 identified intracellular, cytoplasmic lanthanide storage in an organism that harnesses these metals for its metabolism. Here, we describe the extracellular and intracellular accumulation of lanthanides in the *Beijerinckiaceae* bacterium RH AL1, a newly isolated and recently characterized methylotroph. Using ultrathin-section transmission electron microscopy (TEM), freeze fracture TEM (FFTEM), and energy-dispersive X-ray spectroscopy, we demonstrated that strain RH AL1 accumulates lanthanides extracellularly at outer membrane vesicles (OMVs) and stores them in the periplasm. High-resolution elemental analyses of biomass samples revealed that strain RH AL1 can accumulate ions of different lanthanide species, with a preference for heavier lanthanides. Its methanol oxidation machinery is supposedly adapted to light lanthanides, and their selective uptake is mediated by dedicated uptake mechanisms. Based on transcriptome sequencing (RNA-seq) analysis, these presumably include the previously characterized TonB-ABC transport system encoded by the *lut* cluster but potentially also a type VI secretion system. A high level of constitutive expression of genes coding for lanthanide-dependent enzymes suggested that strain RH AL1 maintains a stable transcript pool to flexibly respond to changing lanthanide availability. Genes coding for lanthanide-dependent enzymes are broadly distributed taxonomically. Our results support the hypothesis that central aspects of lanthanide-dependent metabolism partially differ between the various taxa.

**IMPORTANCE** Although multiple pieces of evidence have been added to the puzzle of lanthanide-dependent metabolism, we are still far from understanding the physiological role of lanthanides. Given how widespread lanthanide-dependent enzymes are, only limited information is available with respect to how lanthanides are taken up and stored in an organism. Our research complements work with commonly studied model organisms and showed the localized storage of lanthanides in the periplasm. This storage occurred at comparably low concentrations. Strain RH AL1 is able to accumulate lanthanide ions extracellularly and to selectively utilize lighter lanthanides. The *Beijerinckiaceae* bacterium RH AL1 might be an attractive target for developing biorecovery strategies to obtain these economically highly demanded metals in environmentally friendly ways.

## INTRODUCTION

Lanthanides, also known as rare earth elements (REE), are mistakenly considered rare. The average abundance of lighter lanthanides (La, Ce, Nd) in the Earth’s crust ranges between 60 and 120 ppm, which makes them as abundant (La, Nd) or more abundant (Ce) than common metals, such as copper and zinc (1). The reason why lanthanides are considered rare is that they occur primarily in poorly soluble phosphate and carbonate minerals (2), which impairs their recovery. In addition, they often cooccur, which makes their separation difficult (3). Due to their favorable mechanical, chemical, magnetic, and optical properties, lanthanides are in heavy demand for numerous high-tech applications (3). Examples include permanent magnets, superconductors, optical glass, lasers, and batteries for electric cars.

The ionic radii of the lighter lanthanides (La, Ce, Nd) are similar to that of calcium (1). It was previously hypothesized that lanthanides would be superior cofactors in calcium-dependent enzymes due to their higher valence (i.e., 3+, whereas calcium's is 2+), increased Lewis acidity, and high charge-to-radius ratios (e.g., Ca has a charge of 2+ and ionic radius of 1.00 Å, whereas La has a charge of 3+ and an ionic radius of 1.03 Å) (4). Lanthanum has been dubbed “supercalcium” in the past (5). The evolution of lanthanide-dependent enzymes was, however, considered unlikely due to their poor bioavailability (4). For decades, calcium-dependent methanol dehydrogenases (MDHs) of the Mxa type were considered to catalyze the only pathway for methanol oxidation in methylotrophic microorganisms (6–8). This changed with reports describing that related Xox-type MDHs are stimulated upon lanthanide addition (9–12).

The role of Xox-type MDH in methanol oxidation was questioned at first. Xox-type MDHs are only distantly related to Mxa-type MDHs (<50% amino acid sequence identity), and they lack subunits characteristic of Mxa-type MDHs (13). Genes coding for XoxF were detected in diverse environments (13), and the screening of (meta)genomes showed that *xoxF* genes are widely distributed among taxa. This includes poorly characterized taxonomic groups, such as “*Candidatus* Tectomicrobia” and “*Candidatus* Rokubacteria,” as well as nonmethylotrophic taxa (14–16). Xox-type MDHs are meanwhile considered ecologically more relevant than Mxa-type MDHs, and they are assumed to be of more ancient origin (13). Xox-type and Mxa-type MDHs belong to the family of pyrroloquinoline quinone (PQQ)-dependent alcohol dehydrogenases (ADHs), which make use of a Lewis acidic metal cation as a cofactor. This family comprises more than a dozen subgroups, and most of these quinoproteins are believed to rely on lanthanides as cofactors (13). Among the methylotrophs, we distinguish those that possess both Mxa- and Xox-type MDHs and those that possess only Xox-type MDH. In the case of the former, the expression of the underlying genes is inversely regulated by the so-called lanthanide switch (17). The more widespread occurrence of Xox-type MDH in organisms that at the same time lack Mxa-type MDHs suggests that other regulatory mechanisms that control lanthanide-dependent metabolism must exist.

Although lanthanide utilization is widely studied, our understanding of basic aspects of lanthanide-dependent metabolism is still in its infancy. This includes lanthanide uptake and storage. Recent work with Methylorubrum extorquens AM1 and M. extorquens PA1 has shown that lanthanide uptake is linked to TonB-ABC transport systems and lanthanide-shuttling proteins, such as lanmodulin (18–21), encoded by the *lut* (lanthanide utilization and transport) gene cluster. The involvement of TonB-dependent receptors makes it likely that lanthanide uptake is linked to extracellular chelators, similar to what is known for iron and copper uptake (17). Lanthanides are stored intracellularly in M. extorquens AM1 in mineral form (20). The widespread occurrence of lanthanide-dependent enzymes in (non)methylotrophic organisms implies that multiple mechanisms for sensing, uptake, and storing lanthanides exist in organisms that harness these metals for their metabolism.

In the present study, we use the recently characterized *Beijerinckiaceae* bacterium RH AL1 (22) to obtain additional insights into lanthanide-dependent metabolism, with a focus on lanthanide uptake and storage. Strain RH AL1 was isolated from early-industrial soft-coal slags enriched in bioavailable lanthanides, possesses multiple lanthanide-dependent PQQ ADHs, and lacks an Mxa-type MDH (22, 23). We combined ultrathin-section transmission electron microscopy (TEM) and freeze fracture TEM (FFTEM) with electron-dispersive X-ray spectroscopy (EDX) to study cultures grown with different lanthanum concentrations. We show that lanthanides are accumulated extracellularly at outer membrane vesicles (OMVs) and that they are stored intracellularly in the periplasm. Elemental analysis of cultures grown with different concentrations of lanthanum or a mixture of lanthanides showed that strain RH AL1 prefers to accumulate heavier lanthanides. Transcriptome sequencing (RNA-seq)-based gene expression profiling showed a constitutive expression of genes coding for lanthanide-dependent enzymes and that potential uptake mechanisms are downregulated if lanthanides are present in excess.

## RESULTS

### Lanthanide concentration and extracellular lanthanide accumulation.

**FIG 1:**
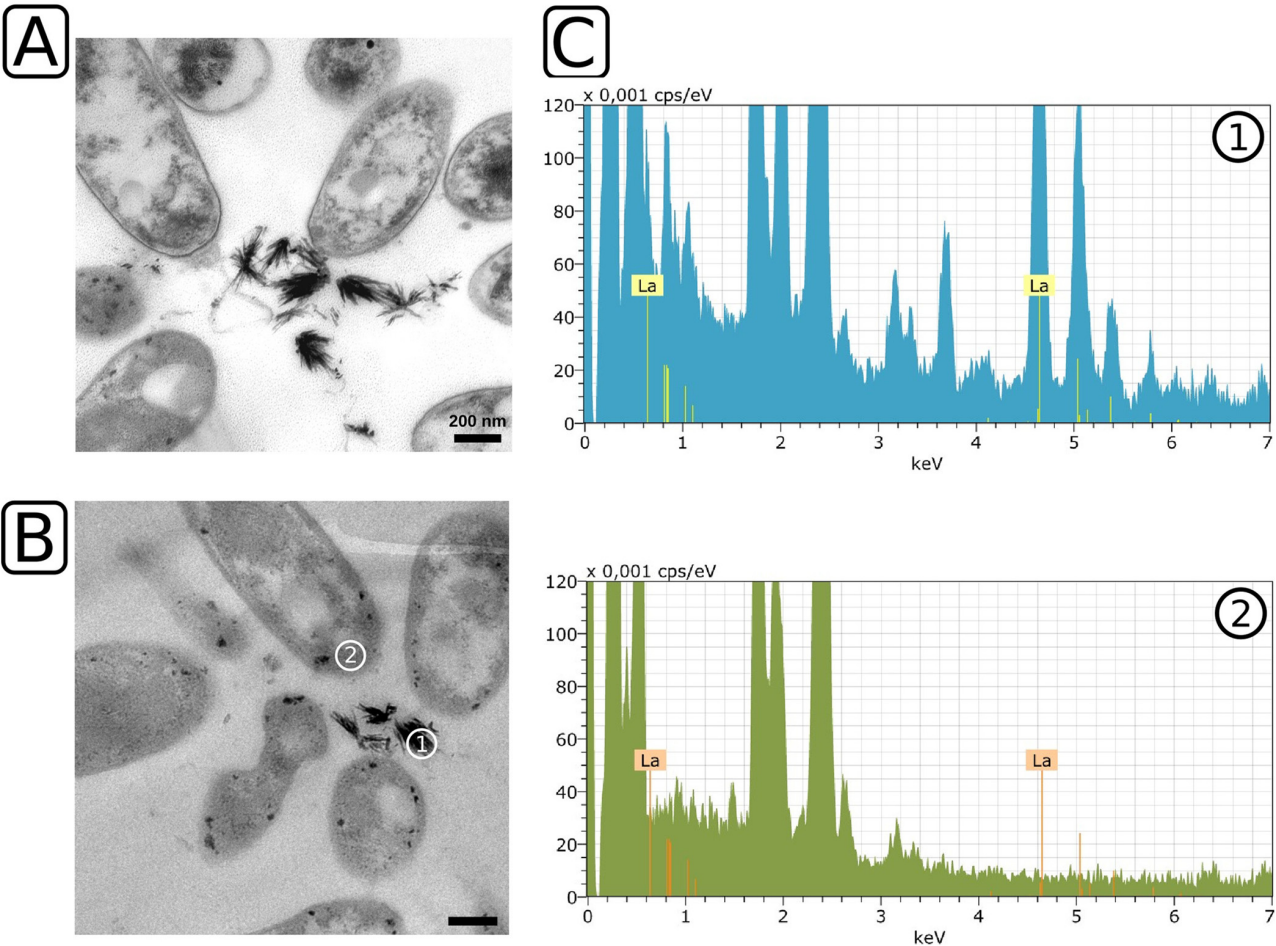
Ultrathin-section transmission electron microscopy and EDX analysis of *Beijerinckiaceae* bacterium RH AL1 grown with 10 μM lanthanum. Strain RH AL1 was grown in MM2 medium (pH 5 to 5.5) with methanol (1% [vol/vol]) as the carbon source. Harvested biomass was fixed with glutaraldehyde (2.5% [vol/vol]), dehydrated with ethanol, and stained with uranyl acetate (2% [wt/vol]). Embedded samples were cut and ultrathin sections stained with lead nitrate. (A) Transmission electron micrographs were inspected with a Zeiss CEM 902 A electron microscope (Carl Zeiss AG, Oberkochen, Germany). (B and C) Representative sample areas for lanthanum crystals (area 1) and background signals (area 2) (B) were used for EDX analysis (C) with a Tecnai G2 electron microscope (FEI, Eindhoven, Netherlands). Lanthanum was detected by a multipoint-EDX analysis of the sample areas using an energy-dispersive X-ray spectrometer system, Quantax 200, with an XFlash detector (model 5030; Bruker, Berlin, Germany). Scale bar = 200 nm. keV, kilo electron-volt; cps/eV, counts per second per electron volt. The X-ray energy for lanthanum is highlighted (La, 4.65 keV).

We cultivated strain RH AL1 with either 1 μM or 10 μM lanthanum and methanol as the carbon source to test whether it is able to store lanthanides intracellularly in a concentration-dependent manner. Differences in appearance in response to increased lanthanum supplementation were assessed by ultrathin-section transmission electron microscopy (TEM). When the strain was grown with 10 μM lanthanum, we noted the formation of extracellular, up to 200-nm-long crystal-like structures (Fig. 1A). We found that up to 30.64% of the cells were arranged in clusters around these crystals (average, 28.61% ± 1.75% of counted cells; 3 areas of 484 μm^2^) (see Fig. S1 in the supplemental material). In order to determine whether these mineral-like, crystalline structures were composed of lanthanum, we used energy-dispersive X-ray spectroscopy (EDX) for elemental analysis. We selected representative crystals (Fig. 1B, area 1) and the peripheral areas of cells for comparison (Fig. 1B, area 2). The EDX analysis revealed a pronounced lanthanum signal in the crystal. No lanthanum was detected in the interior of cells that were picked as references (Fig. 1C).

**FIG 2:**
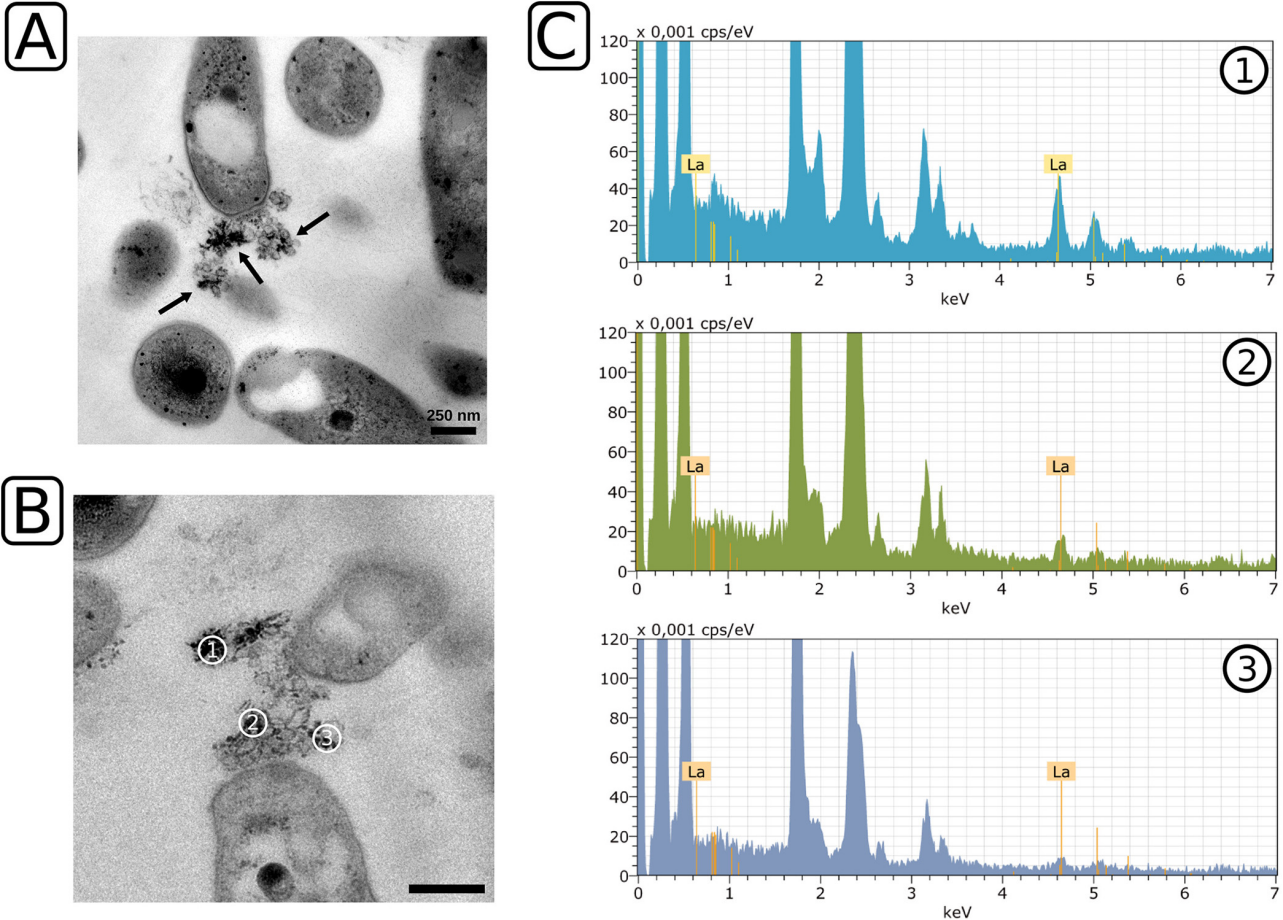
Ultrathin-section transmission electron microscopy and EDX analysis of *Beijerinckiaceae* bacterium RH AL1 grown with 1 μM lanthanum. (A) Transmission electron micrographs were inspected. (B and C) Representative sample areas (B) were used for EDX analysis (C). Details are the same as for Fig. 1. Black arrows indicate small crystalline structures present on the surfaces of OMVs. Scale bar = 250 nm.

Comparable mineral-like structures were not observed when strain RH AL1 was grown with 1 μM lanthanum. Instead, we noticed the presence of OMVs (Fig. 2A; Fig. S2). As with the pattern observed for the crystalline deposits seen at 10 μM lanthanum, cells arranged themselves around the OMVs (average, 23.16 ± 1.75% of counted cells; 3 areas of 484 μm^2^) (Fig. S1). A closer inspection revealed small crystals attached to the released material (Fig. 2A, black arrows). We determined the number of cell clusters per area and observed that 66.2% of all cell clusters featured crystalline structures in contact with OMVs (Fig. S1). To investigate if these small crystals represent smaller accumulations of lanthanides, we repeated the EDX analysis. We selected an area that featured OMVs and small crystalline structures (Fig. 2B, areas 1 to 3). We observed various degrees of lanthanum enrichment (Fig. 2C, areas 1 to 3). The degree of enrichment correlated with the presence of crystalline structures in the measuring field defined for EDX analyses.

**FIG 3:**
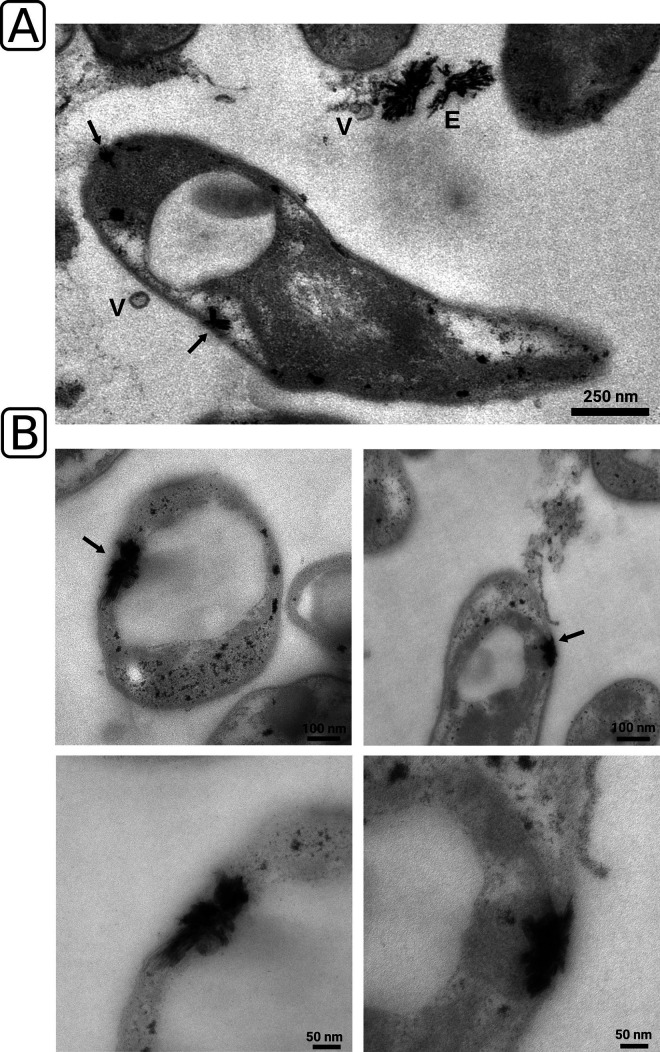
Ultrathin-section transmission electron microscopy and screening for intracellular lanthanum deposits. (A and B) Lanthanum deposits were identified based on morphology and more closely inspected (B, lower panel) for proximity to the cytoplasmic and outer membrane. Black arrows indicate crystalline accumulations. E, extracellular crystalline accumulations; V, outer membrane vesicle.

We additionally screened the ultrathin sections for signs of intracellular lanthanide storage. Occasionally, we were able to spot similar crystalline structures inside cells of RH AL1 (Fig. 3A, black arrows). TEM micrographs revealed that the crystals tend to form cap-like structures close to the cytoplasmic and outer membranes and in proximity to the polar polyhydroxybutyrate vacuoles, characteristic of members of the *Beijerinckiaceae* (Fig. 3B). A clear distinction between the cytoplasmic and outer membranes was not possible based on TEM. The analyzed data suggested that these crystalline deposits might localize to the periplasm but not to the cytoplasm.

### Peripheral and periplasmic storage of lanthanides.

Ultrathin sections for TEM analyses provide limited information about three-dimensional structure, since they represent only two-dimensional layers of embedded material. We used freeze fracture transmission electron microscopy (FFTEM) to bypass this restriction. FFTEM is advantageous, as it provides detailed ultrastructural views of cellular topography and it facilitates the recovery of three-dimensional membrane details.

**FIG 4:**
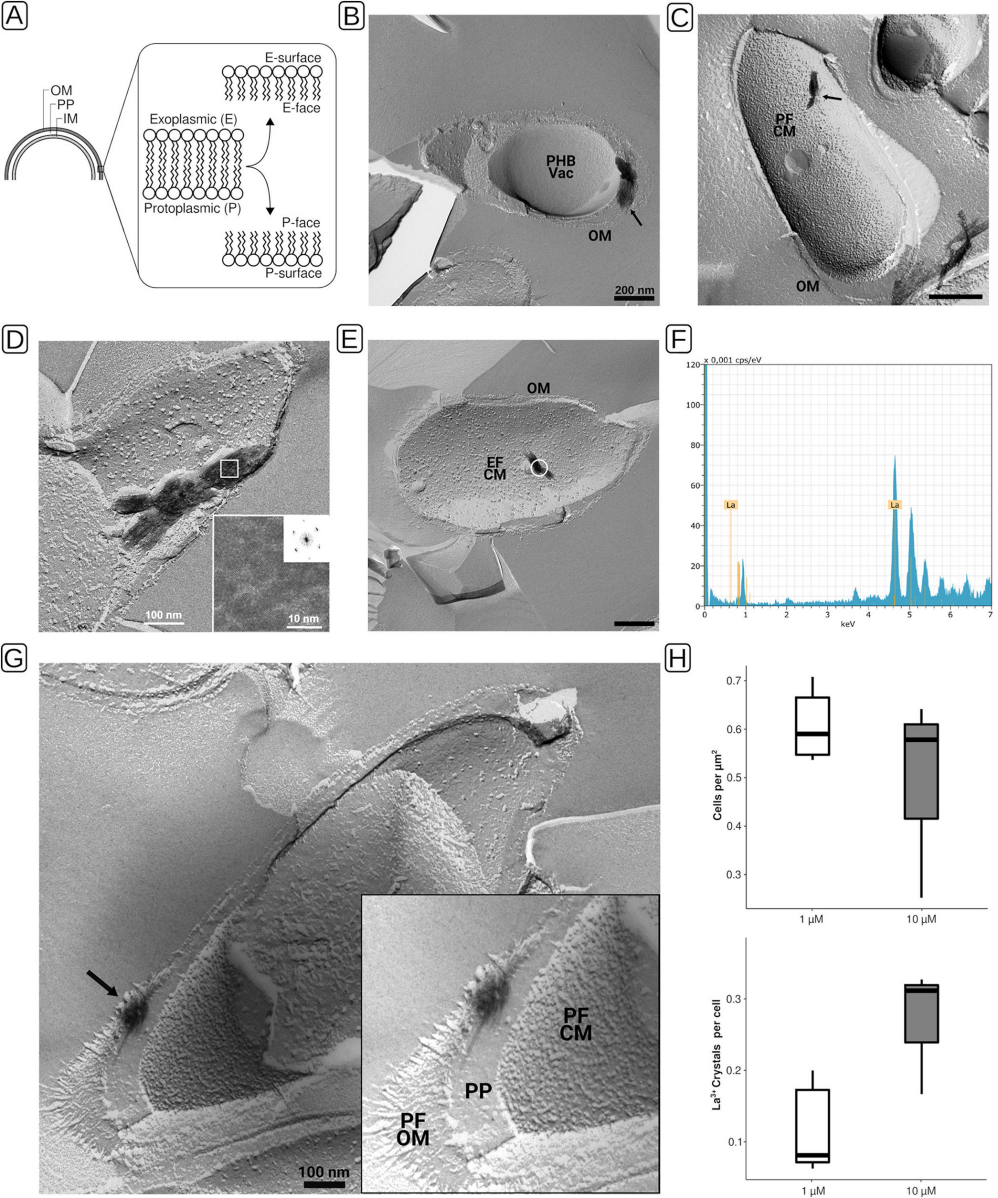
Freeze fracture transmission electron microscopy (FFTEM), EDX analysis, and quantitation of lanthanum deposits of *Beijerinckiaceae* bacterium RH AL1 cultures grown with lanthanum. Biomass was rapidly frozen in a liquid ethane-propane mixture before freeze fracturing was done at −150°C in a BAF400T freeze fracture unit (BAL-TEC, Liechtenstein). Fractured samples were shadowed with 2 nm Pt/C (platinum/carbon), followed by perpendicular evaporation of a 15- to 20-nm-thick carbon layer. (A) Schematic for distinction of fracture faces in FFTEM micrographs. (B to E, G) Freeze fracture replicas were cleaned with commercial sodium hypochlorite solution before being transferred onto uncoated EM grids for examination by TEM. Black arrows indicate crystals that were found in proximity to the cytoplasmic and outer membranes (B to E) and in the periplasmic space (G). (B, D, E, G) TEM micrographs originating from cultures grown with 1 μM lanthanum. (C) TEM micrograph originating from a culture grown with 10 μM lanthanum. (D) The frequency spectra of crystals were calculated by fast Fourier transformation from digital images of frozen and fractured cells. (E) The sample area for EDX analysis is marked with a white circle. (F) The analysis was carried out as outlined for Fig. 1. (H) Lanthanum deposits were counted to determine the number of crystals per cell. For 1 μM lanthanum cultures, a total area of 233 μm^2^ (*n* = 5 areas of 46.6 μm^2^) was analyzed, and for 10 μM lanthanum cultures, a total area of 285.3 μm^2^ (*n* = 3 areas of 95.1 μm^2^) was analyzed. OM, outer membrane; PP, periplasm; IM, inner membrane; CM, cytoplasmic membrane; PF, P face; EF, E face; PHB, polyhydroxybutyrate; Vac, vacuole.

For FFTEM, samples are rapidly frozen and fractured, and the resulting fracture faces are replicated by platinum/carbon evaporation for subsequent inspection by TEM. Fracturing tends to occur along the hydrophobic core of lipid bilayers. In FFTEM micrographs, one distinguishes protoplasmic (P) and exoplasmic (E) fracture faces (F). The P faces of the cytoplasmic and outer membranes are visible in convexly fractured cells, and the E faces are visible in concavely fractured cells (Fig. 4A).

FFTEM samples originating from RH AL1 cultures grown with 1 μM and 10 μM lanthanum revealed electron-dense deposits (Fig. 4B and C, black arrows), similar to the crystalline structures seen with ultrathin-section TEM (Fig. 2 and 3). The sizes of the deposits varied. We observed lengths up to and longer than 200 nm. As already seen with TEM, we detected that the deposits localized to the cell periphery, somewhat close to the cell poles. We occasionally found intracellular deposits outside the cells (Fig. S3, black arrow). In these cases, we could identify imprints in underlying membrane structures, which indicated that the structures were previously in contact with these membranes and presumably quarried out as a result of freeze fracturing. By means of high-resolution imaging (Fig. 4D, inset) and by calculating the frequency spectra of observed deposits via fast Fourier transformation, we could identify an atomic lattice with 0.35-nm and 0.45-nm periodicities, which confirmed the crystalline nature of the identified electron-dense deposits. We performed EDX analysis (Fig. 4E and F) and could show that these crystals are composed mostly of lanthanum (55.6% ± 5.6%).

The observed peripheral accumulation of lanthanum prompted us to generate tilt series of freeze fracture replicas to win additional insights about the three-dimensional ultrastructure of strain RH AL1 with respect to the identified lanthanum deposits. The three generated tilt series (Movies S1 to S3) provided additional support that RH AL1 accumulates lanthanum in the cell periphery in close proximity to the cytoplasmic and outer membranes. Screening-generated FFTEM micrographs revealed one convex cell fracture that allowed us to distinguish the cytoplasmic and outer membranes, which thus confirmed the periplasmic localization of the lanthanum deposits (Fig. 4G). Quantifying the number of lanthanide crystals per cell (Fig. 4H) showed that the number of crystals increased with increasing lanthanum concentration (averages, 1 μM, 0.14 crystal per cell, and 10 μM, 0.27 crystal per cell). Cell densities were comparable between the two investigated concentrations of lanthanum.

### Lanthanide analysis of whole cells.

The extracellular and intracellular accumulation of lanthanides by strain RH AL1 led us to quantify the contents of lanthanide ions in biomass samples by high-resolution elemental analysis. Results from elemental analysis and quantitative PCR (qPCR) were used to determine the average number of lanthanide ions per cell.

**FIG 5:**
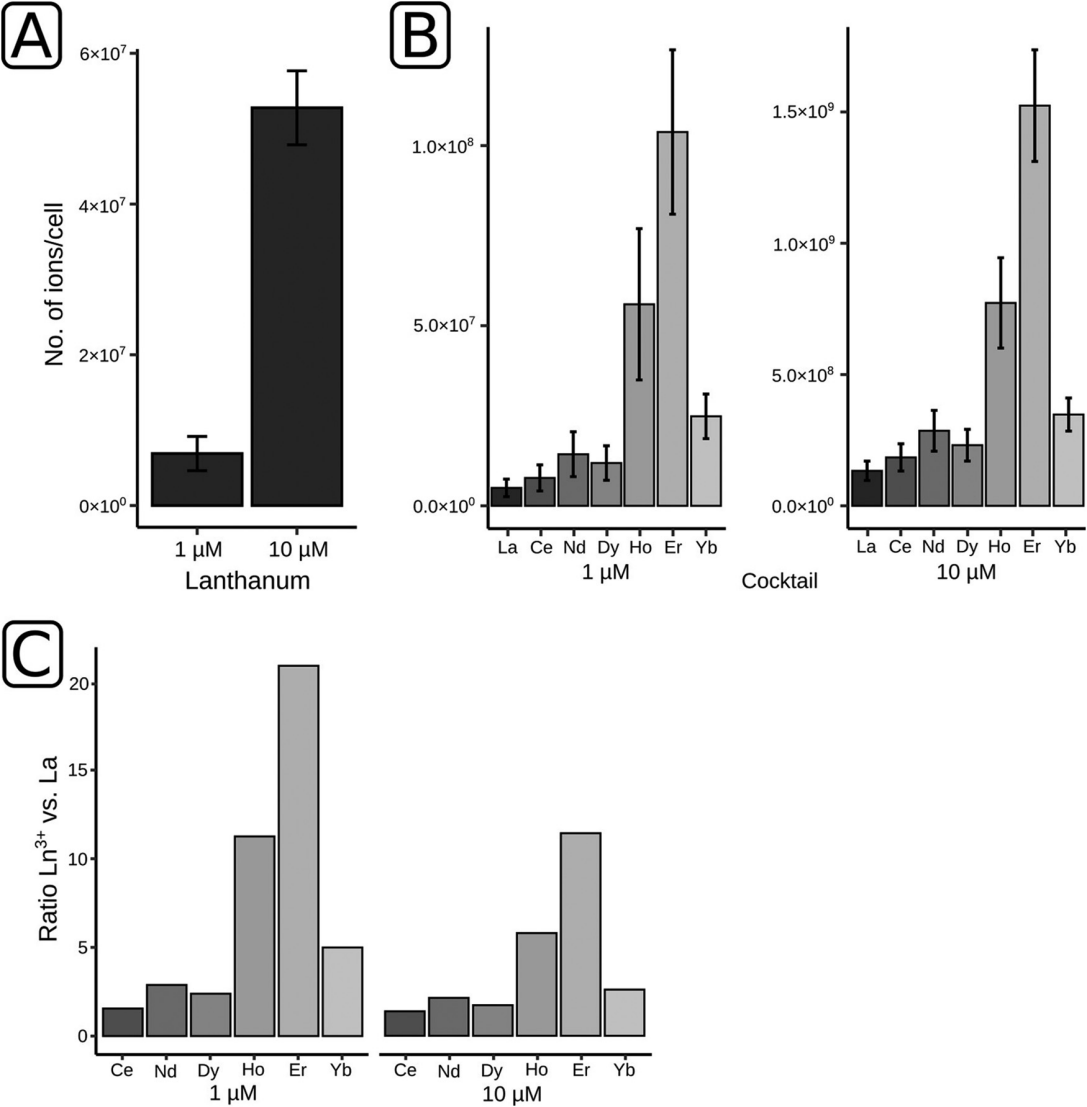
Lanthanide analysis of *Beijerinckiaceae* bacterium RH AL1. Strain RH AL1 was grown with different concentrations (1 and 10 μM) of either lanthanum by itself or a cocktail of lanthanides (La, Ce, Nd, Dy, Ho, Er, Yb). The lanthanide content in harvested biomass was determined by ICP-QqQ-MS with three biological replicates. (A and B) The numbers of lanthanide ions per cell were calculated by linking the lanthanide content of biomass samples with the number of cells present in these samples. Cell numbers were determined by 16S rRNA gene-targeting quantitative PCR with three biological replicates and three technical replicates. Error bars reflect the standard deviation of the ICP-QqQ-MS measurements. (C) Ln^3+^-to-La ratios were calculated by dividing the respective values for ions per cell.

We set up triplicate cultures of RH AL1 supplemented only with lanthanum or with a cocktail of lanthanides (La, Ce, Nd, Dy, Ho, Er, Yb) by applying concentration of either 1 or 10 μM. Based on qPCR, cell numbers ranged between 1.1 × 10^8^ and 3.7 × 10^8^ cells ml^−1^ (Table S1). When grown with 1 μM lanthanum, cells of RH AL1 bound, on average, 1.07 × 10^6^ ions cell^−1^, as analyzed by inductively coupled triple-quadrupole mass spectrometry (ICP-QqQ-MS). At 10 μM, the number of ions was 10 times higher (1.02 × 10^7^ ions cell^−1^) (Fig. 5A; Table S2). The cultures grown with the lanthanide cocktail showed a preferential accumulation of higher-mass lanthanides (Fig. 5B; Table S2). The number of ions ranged between 8.17 × 10^5^ and 2.35 × 10^6^ and between 7.17 × 10^7^ and 1.54 × 10^8^ ions cell^−1^ for La, Ce, and Nd at 1 and 10 μM, respectively. For Ho, Er, and Yb, the numbers were significantly higher. At 1 μM, they ranged between 4.08 × 10^6^ and 1.70 × 10^7^ ions cell^−1^, and at 10 μM, they ranged between 1.87 × 10^8^ and 8.19 × 10^8^ ions cell^−1^. These strong differences became more apparent by calculating the individual ratios of lanthanide ions to lanthanum (Fig. 5C). These ratios were below 3 for the lower-mass lanthanides (Ce, Nd, Dy) and reached values up to 21 and 11 for Er at 1 μM and 10 μM, respectively.

### Differential gene expression induced by lanthanides in heterotrophically grown cultures.

**FIG 6:**
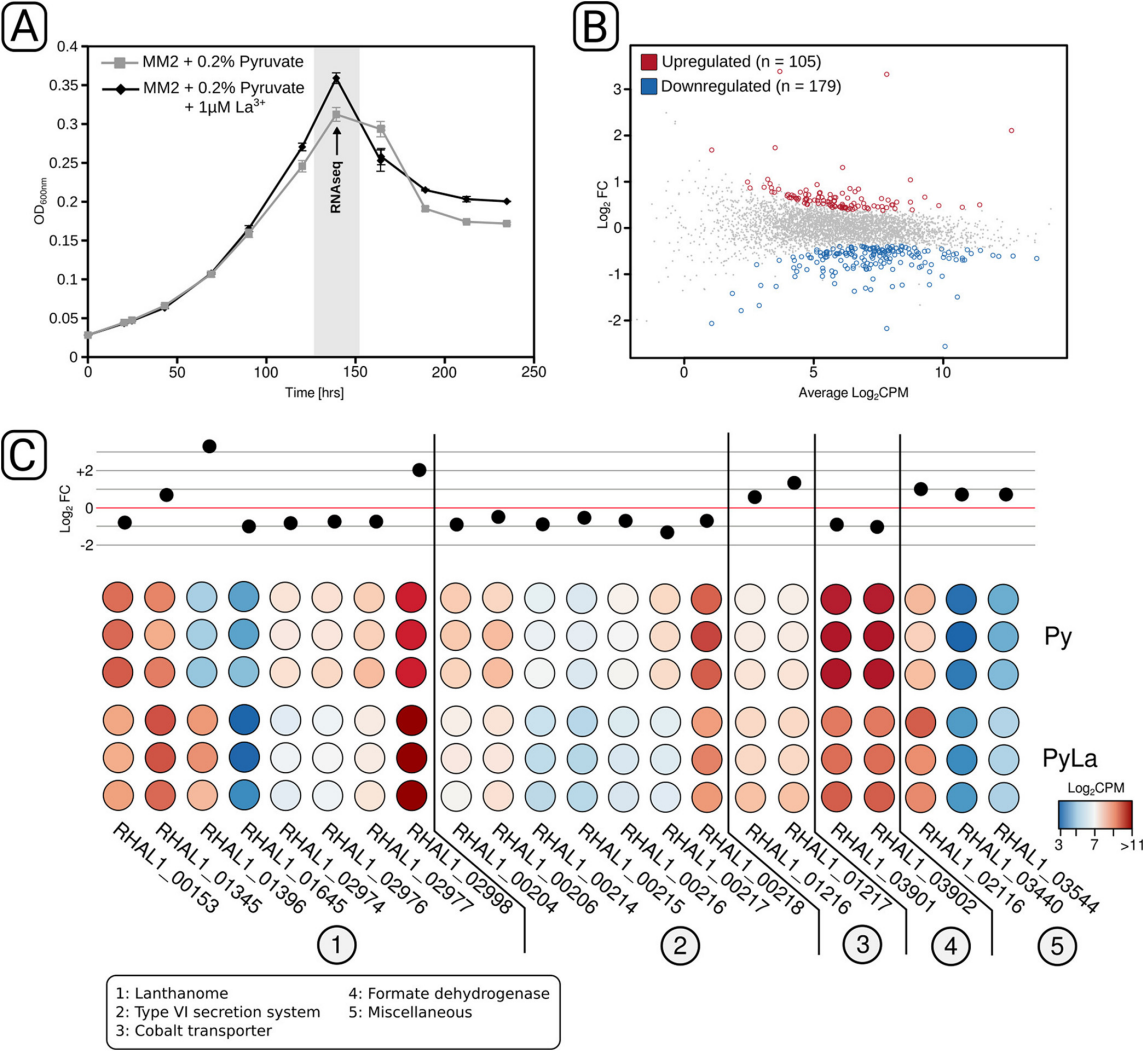
RNA-seq analysis of *Beijerinckiaceae* bacterium RH AL1 grown with and without lanthanum under heterotrophic conditions. Pyruvate was used as a carbon source for cultivation in the MM2 medium. (A) Lanthanum supplementation led to higher maximum optical densities. OD_600nm_, optical density at 600 nm. (B) Biomass samples after 6 days (144 h, marked in gray) of incubation were harvested and used for RNA-seq library preparation. RNA-seq-based, differential gene expression analysis revealed 284 differentially expressed genes. (C) Fold changes and counts per million are shown for selected differentially expressed genes. The red line in the upper half represents no fold change. The color code of the heatmap indicates counts per million. Genes are grouped according to the functions of proteins for which they code. A list with all differentially expressed genes and their annotation can be found in Table S4 in the supplemental material. Log_2_FC, log_2_ fold change, Log_2_CPM, log_2_ counts per million.

RH AL1 cultures grown heterotrophically with pyruvate as the carbon source were used to identify genes that respond to lanthanum supplementation and that might be involved in lanthanide uptake. We noted that the addition of lanthanum had a positive effect on fitness when pyruvate was used as a carbon source (Fig. 6A). With lanthanum, the maximally reached optical density (0.359 ± 0.009) was significantly higher (Student's *t* test, *P* < 0.05) than that of cultures without added lanthanum (0.312 ± 0.007).

We used this phenotypic readout as the starting point for RNA-seq-based differential gene expression analysis. A total of 284 genes were differentially expressed (*P* < 0.05 [false-discovery rate {FDR} corrected]) in response to lanthanum supplementation (Fig. 6B), which is equivalent to 6.7% of the genes encoded in the genome of strain RH AL1. Of these, 179 genes (4.2% of the genes encoded in the genome) were down- and 105 genes (2.5% of the genes encoded in the genome) were upregulated. A closer look at the differentially expressed genes revealed a high proportion of genes coding for (conserved) hypothetical proteins and proteins of unknown function (Tables S3 and S4). The upregulated genes included multiple genes linked to lanthanide-dependent metabolism (Fig. 6C), coding, for instance, for the periplasmic lanthanide-binding protein lanmodulin (RHAL1_01396), XoxF (RHAL1_02998), and the broad substrate alcohol dehydrogenase ExaF (RHAL1_01345). The lanmodulin gene was in fact the most upregulated gene (log_2_ fold change [log_2_FC], 3.3). Comparing the two PQQ ADH genes revealed that *xoxF* responded more strongly (log_2_FC, 2.1) to lanthanum supplementation than *exaF* did (log_2_FC, 0.7). We also observed an upregulation of cobalt transporter genes (RHAL1_01216, RHAL1_01217) and of genes coding for a zinc finger protein (RHAL1_02116), a DNA uptake protein (RHAL1_03544), and a porin (RHAL1_03440).

Genes linked to lanthanide-dependent metabolism were partially also downregulated (Fig. 6C), including the genes coding for homologs of LutH (RHAL1_00153), LutAB (RHAL1_02976, RHAl1_02977), and LutFG (RHAL1_02973, RHAL1_02974). All these genes are linked to the postulated TonB-ABC transport system for lanthanide uptake. The corresponding log_2_FC values ranged between −0.63 (RHAL1_02973) and −0.89 (RHAL1_02974). In addition, lanthanum supplementation led to the downregulation of a gene cluster (RHAL1_00204 to RHAL1_00206, RHAL1_00214 to RHAL1_00218) coding for a type VI secretion system and to a reduced expression of formate dehydrogenase genes (RHAL1_03902, RHAL1_03903).

## DISCUSSION

Lanthanide uptake is central to our understanding of lanthanide-dependent metabolism. Different organisms, including Myxococcus xanthus and Pseudomonas aeruginosa are able to adsorb lanthanides onto their cell surfaces (24, 25). An intracellular storage of europium in the cytoplasm was observed in Thermus scotoductus SA-01 (26). All these observations were made at rather high concentrations (in the millimolar range), and none of these organisms is known to depend on lanthanides.

Our findings provide insights into lanthanide accumulation and uptake in an organism that needs these metals for methylotrophy. We propose that OMVs might serve as an accumulation surface for lanthanides to facilitate uptake. The ability of bacteria to adsorb lanthanides, as well as other metals, is attributed to cell wall and membrane characteristics, especially the presence of phosphoryl and carboxyl groups (27–29), which are enriched in lipopolysaccharides in the outer membrane of Gram-negative bacteria (30). It is known that fine-grained mineral structures can develop on outer membrane surfaces (31). An accumulation on outer membrane surfaces would be rather unspecific and does not explain the localized occurrence of extracellular lanthanum deposits close to the cell poles in the proximity of OMVs. We did not detect any lanthanide accumulation on the outer membrane surface.

OMVs have been known to be a common feature of Gram-negative bacteria for many years, and they have attracted a lot of interest in the context of pathogenesis. OMVs were shown to play a role with respect to virulence factor delivery (32). Functional roles in nonpathogens include cellular communication, surface modification, and the elimination of unwanted metabolites (33). It is meanwhile accepted that OMV biogenesis is a tightly regulated secretion process (34–36). OMVs bud off from the outer membrane of Gram-negative bacteria. OMV biogenesis is controlled by various factors, including locally reduced cell wall integrity (37) and the local accumulation of metabolites and envelope components (38). The cargo of OMVs includes lipids, envelope components, and membrane, periplasmic, and cytoplasmic proteins (39, 40). Mechanisms controlling the protein cargo of OMVs are poorly understood. Studies of Neisseria meningitidis and Porphyromonas gingivalis have shown that OMVs from these organisms are enriched in proteins that are linked to iron and zinc uptake (41, 42). The protein cargo is one important factor controlling the biological role of OMVs. An involvement in lanthanide accumulation would make it necessary for the OMVs of strain RH AL1 to carry lanthanide-binding proteins, such as the periplasmic lanthanide shuttle lanmodulin. A potential role of OMVs in lanthanide uptake is intriguing, and future work is planned for a deeper understanding about the characteristics and protein cargo of OMVs in *Beijerinckiaceae* bacterium RH AL1.

Unlike with the model methylotroph M. extorquens AM1, no detectable cytoplasmic lanthanide stores were detected in the *Beijerinckiaceae* bacterium RH AL1. Instead, RH AL1 stores lanthanides intracellularly but apparently mostly in the periplasm and at low concentrations. Periplasmic accumulation might be advantageous, as the maturation and activity of lanthanide-dependent PQQ ADH are supposed to happen in the periplasm (13). Periplasmic storage of lanthanides in M. extorquens AM1 was seen only in mutants that lack components of the postulated TonB-ABC transport system for lanthanide uptake (17). Mutants lacking the ABC transporter for lanthanide uptake from the periplasm into the cytoplasm showed periplasmic lanthanum deposits similar to the ones seen in the *Beijerinckiaceae* bacterium RH AL1 (Fig. 3).

It was postulated for M. extorquens AM1 that lanthanum is complexed with polyphosphate (20) in the cytoplasm. Polyphosphate granules or acidocalcisomes are among the few known, subcellular, membrane-surrounded structures in bacteria (43). Acidocalcisomes are linked to functions other than polyphosphate storage, including cation sequestration, especially of calcium (43, 44). Different metal cations can be stored in acidocalcisomes, including copper, zinc, and iron (45–47). We did not detect a strong phosphorus signal in the EDX data of the identified lanthanide deposits in strain RH AL1, suggesting a different lanthanide storage mechanism in RH AL1. The exact nature of the periplasmic deposits remains an open question for now.

Our metallomics data showed that the *Beijerinckiaceae* bacterium RH AL1 can accumulate ions of different lanthanide species, with a preference for heavier lanthanides. The generally low bioavailability of lanthanides and the previous identification of a TonB-ABC transport system in M. extorquens (18–20) pointed toward a chelator-based uptake. Iron and copper homeostasis in methylotrophs relies on high-affinity chelators known as siderophores (iron) and chalkophores (copper) (17), but until now, no lanthanide metallophore has been described. It is unclear whether all lanthanide-utilizing microbes possess and need such lanthanophores. *Methylacidiphilum fumariolicum* SolV was isolated from an acidic (pH 1 to 2) volcanic mudpot with high loads of solubilized lanthanides (in the micromolar range) (12), making dedicated lanthanide metallophores presumably less important. M. extorquens AM1 secretes a high-affinity, lanthanide-binding metallophore with a preference for lighter lanthanides (48). Strain RH AL1, isolated from acidic (pH 3.4 to 3.6) and metal-rich slags that contain bioavailable, lighter lanthanides (La, Ce, Nd; 27 to 64 ppm) (22), might be adapted and has a reduced need for a high-affinity lanthanide uptake system. Until now, we have no evidence that strain RH AL1 produces and secretes lanthanophores. We previously showed that only lighter lanthanides (La, Ce, Nd) promote the growth (22) of RH AL1 with methanol.

Work with *M. fumariolicum* SolV showed that the structural differences in XoxF that are dependent on the bound lanthanide cofactor are negligible (49). The catalytic properties are, however, altered due to differences in the ionic radii and coordination number. A study of M. extorquens AM1 revealed that the increasing Lewis acidity of lanthanides with increasing mass impairs the electron transfer from XoxF. XoxF transfers the electron released upon methanol oxidation to XoxG, a cytochrome *c*_L_. A biochemical characterization of XoxG has shown that the reduction potential of XoxG is fine-tuned based on the lanthanide species present in its XoxF counterpart (50). The uptake of heavier lanthanides and their incorporation in XoxF would likely disturb methanol oxidation in strain RH AL1. If it is adapted to lighter lanthanides, as suggested by previous incubation experiments, the reduction potential of its XoxG would not facilitate electron extraction from XoxF if heavier lanthanides are incorporated as a metal cofactor. The idea that the redox properties of XoxG are fine-tuned based on the lanthanides found in their XoxF counterparts was supported by the finding that *xoxG* genes from other genera cannot complement an *xoxG* deletion in *Methylomonas* sp. strain LW-13 (51). RH AL1 grown with lanthanum and the lanthanide cocktail did not show any difference in growth. A selective uptake might operate to prevent the heavier lanthanides from distorting the methanol oxidation machinery. Our previous genomic analysis revealed gene products homologous to the *lut* cluster (22). The degree of homology to M. extorquens AM1 was rather low (LutH homolog, 37% amino acid sequence identity). Considering that we did not see cytoplasmic lanthanide deposits in RH AL1, the homologs of the *lut* cluster-encoded ABC transporter might have another function in our strain.

The presence of lanthanide-dependent PQQ ADH in diverse taxonomic groups, including taxa not known to be involved in methylotrophy (14–16), suggests that lanthanide-dependent metabolism is functionally broader. Additional lanthanide uptake mechanisms might also exist in the characterized TonB-ABC transport system. Porin proteins in RH AL1 have been postulated based on the high expression of porin-encoding genes under methylotrophic growth conditions (22), which is supported by the observed upregulation of a gene coding for a porin in response to lanthanum addition in this study.

The lack of genetic tools available for our strain limits the identification of additional genes and gene products linked to lanthanide-dependent metabolism. Our RNA-seq-based gene expression analysis made use of the fitness increase upon lanthanide supplementation under heterotrophic growth conditions with pyruvate as the carbon source. The observed, slight downregulation of the genes coding for homologs of the TonB-ABC transport system was in line with previous reports of M. extorquens AM1 and *Methylotuvimicrobium buryatense* 5GB1C that observed a downregulation of this machinery in response to excess lanthanides (20, 52). Similar observations are known from iron homeostasis (53, 54). Based on our RNA-seq data, *xoxF* and *exaF* are constitutively expressed in the absence of methanol and upregulated in the presence of lanthanides. The constitutive expression of these genes under nonmethylotrophic conditions was unexpected, as constitutive gene expression is energetically expensive and does not allow switching between adapted phenotypes under different environmental conditions (55). Constitutive expression allows organisms to fine-tune their gene expression, allowing suboptimal growth at all times without any adaptation lag (55). We hypothesize that the constitutive gene expression of *exaF* and *xoxF* allows RH AL1 to flexibly respond to the changing availability of lanthanides in the environment.

Our data showed that the gene coding for the periplasmic lanthanide shuttle protein lanmodulin is highly inducible by lanthanide supplementation. The genome of RH AL1 contains a gene cluster coding for a type VI secretion system (T6SS), which was downregulated when lanthanides were added. T6SSs are commonly considered bacterial weapons that are associated with the translocation of effector proteins into target cells (56, 57). T6SSs can also partake in metal uptake. Burkholderia cenocepacia releases the metallophore effector TseM via its T6SS for scavenging manganese (58). Yersinia pestis and Burkholderia thailandensis likewise make use of their T6SSs for secreting zinc-binding effectors (58).

### Concluding remarks.

We here demonstrate periplasmic storage of lanthanides in a microbe that needs these metals for its metabolism. Intracellular storage was observed at low micromolar concentrations. *Beijerinckiaceae* bacterium RH AL1 might be an attractive target for developing strategies to recover lanthanides in an efficient and environmentally friendly way. Our electron microscopy, metallomics, and RNA-seq data expanded our understanding of lanthanide uptake. The *Beijerinckiaceae* bacterium RH AL1 is able to accumulate lanthanide ions extracellularly using OMVs. Specific uptake mechanisms, presumably a TonB-ABC transport system first described in M. extorquens but maybe also a type VI secretion system, facilitate the selective uptake of lanthanide ions for which the methanol oxidation machinery is likely tuned. Our RNA-seq analysis showed the constitutive expression of *xoxF* and *exaF* in the absence of lanthanides and under nonmethylotrophic growth conditions. Strain RH AL1 appears to maintain a stable transcript pool for these genes to be able to flexibly respond to lanthanide availability.

## MATERIALS AND METHODS

### Cultivation.

The *Beijerinckiaceae* bacterium RH AL1 was grown in MM2 medium as described previously (59) with either methanol (1%, vol/vol) or pyruvate (0.2%, wt/vol) as the carbon source, and the medium was supplemented with two different concentrations (1 and 10 μM) of lanthanum or a lanthanide cocktail (La, Ce, Nd, Dy, Ho, Er, Yb). All lanthanides were purchased as trichloride salts. Cultivation was done in acid-washed 120- or 200-ml serum bottles with boiled and sterilized butyl rubber stoppers. Biomass was harvested by centrifugation (10,000 × *g*, 10 min, room temperature). Harvested biomass was either processed immediately or stored at −80°C until further usage.

### TEM.

For transmission electron microscopy (TEM), harvested cell material was fixed in 2.5% (vol/vol) glutaraldehyde in cacodylate buffer (100 mM, pH 7.4) for 2 h at 20°C. Fixed samples were washed three times with cacodylate buffer and postfixed with 1% osmium tetroxide in cacodylate buffer for 2 h at 20°C. Samples were further processed as outlined in the supplemental material and as described previously (22). The numbers of cells arranged around released OMVs/vesicular material and lanthanum crystals were determined by counting visible cell sections per area. For 1 μM and 10 μM lanthanum cultures, a total area of 1,452 μm^2^ (*n* = 3 areas of 484 μm^2^) was analyzed. In the case of the 1 μM cultures, the number of cell clusters per area and the proportion of cell clusters with crystalline deposits were determined as well.

### FFTEM.

For freeze fracture transmission electron microscopy (FFTEM), aliquots of pelleted biomass were enclosed between two 0.1-mm-thick copper profiles as used for the sandwich double-replica technique. The sandwiches were physically fixed by rapid-plunge freezing in a liquid ethane/propane mixture and cooled by liquid nitrogen. Freeze fracturing was performed at −150°C in a BAF400T freeze fracture unit (BAL-TEC, Liechtenstein) using a double-replica stage. Details about the further processing of fractured samples are given in the supplemental material. Lanthanum crystals were quantified by counting the cell fractures per area and the lanthanum deposits per cell. For 1 μM lanthanum cultures, a total area of 233 μm^2^ (*n* = 5 areas of 46.6 μm^2^) was analyzed, and for 10 μM lanthanum cultures, 285.3 μm^2^ (*n* = 3 areas of 95.1 μm^2^) was analyzed.

### Tilt series of freeze fracture replicas.

For the recording of tilt series, freeze fracture replicas were placed in a tilt/rotate specimen holder (model 626; Gatan, Pleasanton, CA, USA). Data sets were recorded using a CM120 cryo-transmission electron microscope (FEI, Eindhoven, Netherlands) operated at 120 kV. Images were captured and aligned every 2° over a −60° to +60° range using a 2K CMOS camera (F216, EMMENU V4.0 software; camera and software were from TVIPS, Munich, Germany). Tilt series movies were generated by merging individual images into a. gif file using *gimp* (v. 2.8) (https://www.gimp.org/).

### EDX of ultrathin sections and freeze fracture replicas.

For energy-dispersive X-ray spectroscopy (EDX) analyses, ultrathin sections and freeze fracture replicas were measured using a Tecnai G2 electron microscope (FEI, Eindhoven, Netherlands). High-angle annular dark-field (HAADF) images were acquired at 200 kV, and the electron beam was operated in STEM (scanning transmission electron microscopy) mode. For the detection of lanthanum, a multipoint EDX analysis of the samples was performed by using an energy-dispersive X-ray spectrometer system, Quantax 200, with an XFlash detector (model 5030; Bruker, Berlin, Germany).

### DNA extraction.

Extractions were carried out as described previously (60). The used protocol is comprehensively described in the supplemental material.

### Lanthanide analysis of biomass by triple-quadrupole inductively coupled plasma mass spectrometry (ICP-QqQ-MS).

Biomass samples from cultures grown in medium with different lanthanide concentrations were treated in open vials with 500 μl of a 1:1 mixture of concentrated Suprapur nitric acid (65%, vol/vol; Merck Millipore, Darmstadt, Germany) and hydrogen peroxide (37%; Merck Millipore) at 80°C for 2 h in a thermomixer. This solution was then diluted with ultrapure water to a total volume of 3 ml. Calibration standards were prepared by serially diluting lanthanide standards in 2% nitric acid to concentrations between 0 and 10 mg liter^−1^. Rhodium (Merck Millipore) was added as an internal standard to each sample and each calibration standard at a final concentration of 1 μg liter^−1^ (61).

Samples and calibration standards were analyzed for the lanthanide isotopes ^139^La, ^140^Ce, ^142^Nd, ^164^Dy, ^165^Ho, ^169^Er, and ^172^Yb and the internal standard ^103^Rh with a high-resolution 8800 ICP-QqQ-MS (Agilent Technologies, Waldbronn, Germany) coupled with an Aridus II desolvating c-flow nebulizer system (Teledyne CETAC, Omaha, NE, USA). The tune parameters for ICP-QqQ-MS and desolvating c-flow nebulizer system were optimized for the high sensitivity of target lanthanide isotopes using ICP-QqQ-MS MassHunter 4.2 workstation software (Table S5). Argon sweep gas was set to a flow rate of 2.25 liters min^−1^, and nitrogen gas flow was set to 2 ml min^−1^ in the desolvating c-flow nebulizer system. Hydrogen gas was used as a reaction gas at a flow rate of 3.3 ml min^−1^ in an Octopole reaction system (ORS3) of the ICP-QqQ-MS instrument to reduce polyatomic interferences. All measurements were performed with three biological replicates. The number of lanthanide ions per cell was calculated by correlating the results from elemental analysis with 16S rRNA gene qPCR data. Strain RH AL1 possesses one rRNA operon, and we assumed that one 16S rRNA gene copy is equivalent to one cell.

### Real-time qPCR.

Numbers of 16S rRNA gene copies were determined as a proxy for cell numbers by qPCR using a CFX96 instrument (Bio-Rad, Munich, Germany), Brilliant II SYBR Green QPCR master mix (Agilent Technologies, Germany), and the primer combination Bac8Fmod/Bac338Rabc (62–64). One to 20 ng of genomic DNA was used as a template. Cycling conditions were as follows: 10 min of denaturation at 95°C, followed by 45 cycles of 30 s at 95°C, 30 s at 55°C, 25 s at 72°C, 15 s at 78°C, and 15 s at 80°C. Plasmids with cloned bacterial 16S rRNA gene fragments from freshwater environments were used for constructing standard curves. Standard curves were linear from 5 × 10^8^ to 5 × 10^2^ copies, with *R*^2^ values and PCR efficiencies above 0.99 and 80%, respectively. The presence of PCR inhibitors was assessed by a 10-fold dilution series of samples. The specificity was checked by melt curve analysis.

### RNA extraction and mRNA enrichment.

Biomass was collected by centrifugation and subsequently subjected to RNA extraction based on a method described previously (22, 65). RNA was quantified by fluorometry and its integrity checked by agarose gel electrophoresis. rRNA was depleted by subtractive hybridization using the MICROBExpress bacterial mRNA enrichment kit (Thermo Fisher Scientific, Schwerte, Germany). Successful depletion was assessed by chip-based, high-resolution gel electrophoresis using a Bioanalyzer instrument and RNA 6000 Pico reagents (Agilent). Sequencing libraries were prepared with the NEBNext Ultra II directional RNA library prep kit for Illumina (New England Biolabs, Frankfurt, Germany).

### RNA-seq and data preprocessing.

Equimolarly pooled RNA-seq libraries were sequenced in rapid mode on an Illumina (Munich, Germany) HiSeq 2500 instrument (2 × 150 bp, paired ends). Sequencing was carried out by CeGaT GmbH (Tübingen, Germany). Demultiplexing was carried out with bcl2fastq (v2.19) (Illumina). The quality of raw, demultiplexed RNA-seq data sets was inspected using FastQC (v0.11.7) (66). Quality trimming (settings: minlen = 75, qtrim = rl, ktrim = rl, k = 25, mink = 11, trimq = 20, qtrim = rl) and the removal of still-present adapter sequences were done with BBDuk (v38.26) (67) using the included database of common sequence contaminants and adapters. rRNA-derived sequences, as well as noncoding RNA sequences, were filtered out with SortMeRNA (v2.1) (68) and its precompiled databases SILVA (69) and Rfam (70). The remaining, putatively mRNA-derived sequences were mapped onto the available reference genome of strain AL1 (22, 71) (EBI accession no. LR590083 [genome] and LR699074 [plasmid]) using BBMap (v28.26) (67) (settings: slow, k = 11). The resulting. bam files were sorted and indexed with SAMtools (v1.3.1) (72). Genome annotations were used for generating simplified annotation format files (http://bioinf.wehi.edu.au/featureCounts/) for subsequent read counting. Read counts, meaning the number of mapped reads per feature (e.g., coding genes), were deduced from the generated. bam files using the program featureCounts implemented in Subread (v1.6.3) (73, 74).

### Differential gene expression analysis.

Differential gene expression analysis was done in the R software framework for statistical computing (v3.5.1) (75), using the package edgeR (v3.20.9) (76), including all its dependencies. Starting from merged read count data of the two experimental conditions, pseudo-counts, generated by calculating log_2_(counts + 1), were used for preliminary data exploration by generating MA (mean of the normalized counts versus the log_2_ fold changes for all genes tested) plots and multidimensional scaling plots of gene expression profiles using the plotMDS function of limma (v3.42.0) (77). The biological coefficient of variation was calculated for each gene to assess biology-derived variation within replicate groups. Genes identified to be differentially expressed were false-discovery rate (FDR) corrected and filtered with respect to the log_2_ fold change (log_2_FC), FDR-corrected *P* value, and absolute gene expression in log_2_ counts per million (cpm).

### Data availability.

RNA-seq data sets are available from EBI/ENA under the ArrayExpress submission E-MTAB-9481 (https://www.ebi.ac.uk/arrayexpress/experiments/E-MTAB-9481/).
