# Supplementary material for: Extracellular and Intracellular Lanthanide Accumulation in the Methylotrophic Beijerinckiaceae Bacterium RH AL1
Source: Appl Environ Microbiol. 2021 Jun 11;87(13):e03144-20. doi: 10.1128/AEM.03144-20 (PMC8316094; doi:10.1128/AEM.03144-20)
Supplement: Supplemental file 1 — Figures S1 to S3, legends to supplemental movies and tables, supplemental materials and methods. Download AEM.03144-20-s0001.pdf, PDF file, 2.1 MB [file aem.03144-20-s0001.pdf]

<u>Supplementary material:</u> Extracellular and intracellular lanthanide accumulation in the methylotroph Beijerinckiaceae bacterium RH AL1

Carl-Eric Wegner[1#], Linda Gorniak[1], Martin Westermann[2], Frank Steiniger[2], Rohit Budhraja[3], Lorenz Adrian[4], and Kirsten Küsel[1,4]

[1]Institute of Biodiversity, Aquatic Geomicrobiology, Friedrich Schiller University, Dornburger Str. 159, 07743 Jena, Germany

[2]Electron Microscopy Center, Jena University Hospital, Ziegelmühlenweg 1, 07743 Jena, Germany

[3]Helmholtz Centre for Environmental Research - UFZ, Department Isotope Biogeochemistry, Permoserstraße 15, 04318 Leipzig

[4]Helmholtz Centre for Environmental Research - UFZ, Department Environmental Biotechnology, Permoserstraße 15, 04318 Leipzig

[5]German Center for Integrative Biodiversity Research (iDiv) Halle-Jena-Leipzig, Deutscher Platz 5E, 04103 Leipzig, Germany

# Corresponding author:

Carl-Eric Wegner

Email address: carl-eric.wegner@uni-jena.de

SUPPLEMENTARY MATERIAL

Supplementary Information. Complementary information about used materials and applied methods.

FIG S1. Quantitation of cells of Beijerinckiaceae bacterium RH AL1 arranged in clusters around OMVs/vesicular material (medium supplemented with 1µM lanthanum) and around lanthanum crystals (medium supplemented with 10 µM lanthanum). The proportion of cells arranged in clusters (**A**) and the number of cells per μm^2 (**B**) was determined. In case of 1 µM samples we also determined the number of cell clusters per area and the proportion of cell clusters with crystal-like deposits (**C**). The quantitation was based on counting cells in overview transmission electron micrographs (n = 3 areas of 484 μm^2).

FIG S2. Transmission electron microscopy of Beijerinckiaceae bacterium RH AL1 grown with 1 µM lanthanum. Screening transmission electron micrographs revealed the presence OMVs (**A-C**). Black arrows arrows indicate accumulations of OMV material. V = outer membrane vesicle, scale bar = 250 nm.

FIG S3. Freeze-fracture transmission electron microscopy (FFTEM) of Beijerinckiaceae bacterium RH AL1 grown with 1 µM lanthanum. Inspecting FFTEM micrographs showed occasionally the presence of extracellular lanthanum deposits that appeared to be quarried out of the cell due to freeze-fracturing. Deposits seem to have been in close contact with the outer membrane as indicated by left imprints.

The black arrow indicates the lanthanum deposit. Im = membrane imprint, IM = inner membrane, OM = outer membrane, scale bar = 250 nm.

Movies S1-S3. Animated tilt series of freeze-fractured samples. Freeze-fracture replicas were tilted and images captured and aligned every 2° over a -60° to +60° range.

Table S1. Quantitative PCR data of biomass samples that have been subjected to lanthanide analysis. 16S rRNA gene copies were determined as a proxy for cell numbers using the primer pair Bac8FMod/Bac338Rabc as described in the materials and methods section. Given that Beijerinckiaceae bacterium RH AL1 possesses one rRNA operon we assumed that one gene copy is equivalent to one cell. The results are based on three biological and three technical replicates. Cocktail refers to the used mixture of light and heavy lanthanides, NC = negative control (strain RH AL1 grown with pyruvate as carbon source without added lanthanides), std_dev = standard deviation.

Table S2. Lanthanide ion content of biomass samples. The number of ions per cell was deduced by correlating the data from the lanthanide analysis of biomass samples with carried out quantitative PCR analyses targeting the 16S rRNA gene. Cocktail refers to the used mixture of light and heavy lanthanides, NC = negative control, std_dev = standard deviation.

Table S3. Overview of gene expression data of Beijerinckiaceae bacterium RH AL1 grown with pyruvate as carbon source and supplemented with and without lanthanum. Gene expression is given in log_2 Counts per million (log_2CPM) . PyLa = RH AL1 grown with pyruvate and lanthanum, Py = RH AL1 grown with pyruvate.

Table S4. Overview of differentially expressed genes in Beijerinckiaceae bacterium RH AL1 when grown with pyruvate as the carbon source in response to lanthanum supplementation. log_2FC = log_2 Fold change, log_2CPM = log_2 Counts per million, FDR = false discovery rate.

Table S5. Tune parameters for ICP-QqQ-MS and desolvating c-flow nebulizer system. PFA = perfluoroalkoxy.

<u>**Note:**</u> All supplementary tables are provided in one combined spreadsheet.

SUPPLEMENTARY INFORMATION

Transmission electron microscopy (TEM). Fixed samples were dehydrated in an ascending ethanol series and subsequently stained with 2% (w/v) uranyl acetate in 50% (v/v) ethanol. Araldite resin (Plano, Wetzlar, Germany) was used for embedding samples according to the manufacturer's instructions. Ultrathin sections (70 nm thickness) were cut using an ultramicrotome Ultracut E (Reichert-Jung, Vienna, Austria) and mounted on Formvar-carbon coated 100 mesh grids (Quantifoil, Großlöbichau, Germany). The ultrathin sections were stained with lead nitrate for 10 minutes, examined in a Zeiss CEM 902 A electron microscope (Carl Zeiss AG,

Oberkochen, Germany), and imaged using a TVIPS 1k Fast-Scan CCD-Camera (TVIPS, Munich, Germany). Ultrathin sections and freeze-fracture replicas were examined in a digital Zeiss EM 900 electron microscope (Zeiss, Oberkochen, Germany; digital upgrade by Point Electronic, Halle, Germany) operated at 80 kV. Digitized images were taken with a wide-angle dual-speed 2K CCD camera controlled by the Sharp:Eye base controller and operated by the Image SP software (TRS, Moorenweis, Germany).

Freeze fracture electron microscopy. The fractured samples were immediately shadowed without etching with 2 nm Pt/C (platinum/carbon) at an angle of 35°, followed by perpendicular evaporation of a 15-20 nm thick carbon layer. The evaporation of Pt/C was controlled by a thin-layer quartz crystal monitor; the thickness of the carbon layer was controlled optically. The obtained freeze-fracture replicas were transferred to a "cleaning" solution (commercial sodium hypochlorite containing 12% active Cl_2) for 30 min at 45°C. Then, the replicas were washed four times in distilled water and transferred onto uncoated EM-grids for examination in a TEM. Frequency spectra of crystals were calculated by FFT (Fast Fourier Transformation) from digital images of freeze-fractured cells using the FFT submenu implemented in the ImageJ software package (Ver. 1.52t, NIH, USA, https://imagej.nih.gov/ij/index.html).

DNA extraction. Biomass was resuspended in 1 mL SET buffer (sucrose, TRIS, EDTA), before additional 1.6 mL SET buffer, 350 µL 10% sodium dodecyl sulfate (SDS), and 30 µL 100 mM phenylmethylsulfonyl fluoride (PMSF) in isopropanol were

added. After being incubated for 2 hours at 60 °C with shaking (900 rpm), samples were extracted twice with 2 mL of phenol:chloroform:isoamyl alcohol (25:24:1). After phase separation by centrifugation (10000 x g, 10 min., room temperature), still present cell debris was removed from the pooled phenol-chloroform phases by an additional centrifugation step. DNA was precipitated overnight at -20°C by adding 12 µL glycogen (20 µg/mL, Sigma-Aldrich, Munich, Germany), 1 mL 7.5 M ammonium acetate and 8 mL 100% ethanol, after extraction of the aqueous phase with chloroform:isoamyl alcohol (24:1). DNA was pelleted by centrifugation (16,000 x g, 60 min., 4°C), washed twice with 80% ethanol and air-dried. DNA integrity and quality was checked by agarose gelelectrophoresis and photometry. DNA was quantified by fluorometry and stored at -20°C until further processing.

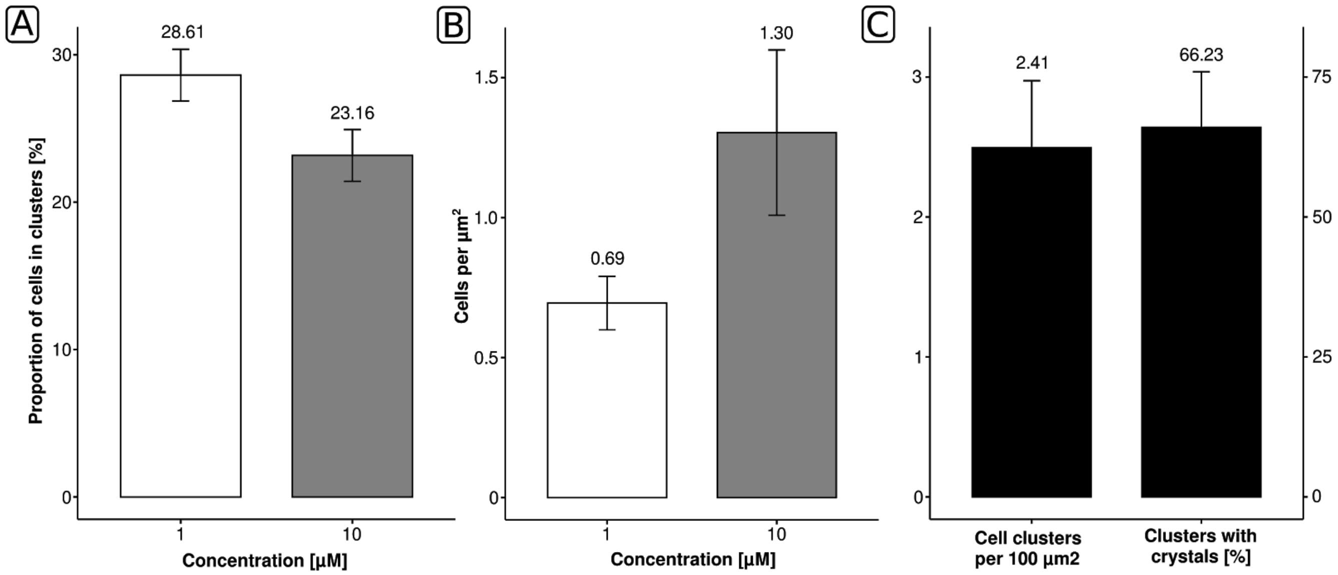
A
28.61
23.16
Proportion of cells in clusters [%]
30
20
10
0
1
10
Concentration [µM]
B
1.30
0.69
Cells per µm2
1.5
1.0
0.5
0
1
10
Concentration [µM]
C
2.41
66.23
3
2
1
0
75
50
25
0
Cell clusters per 100 µm2
Clusters with crystals [%]

Fig. S1

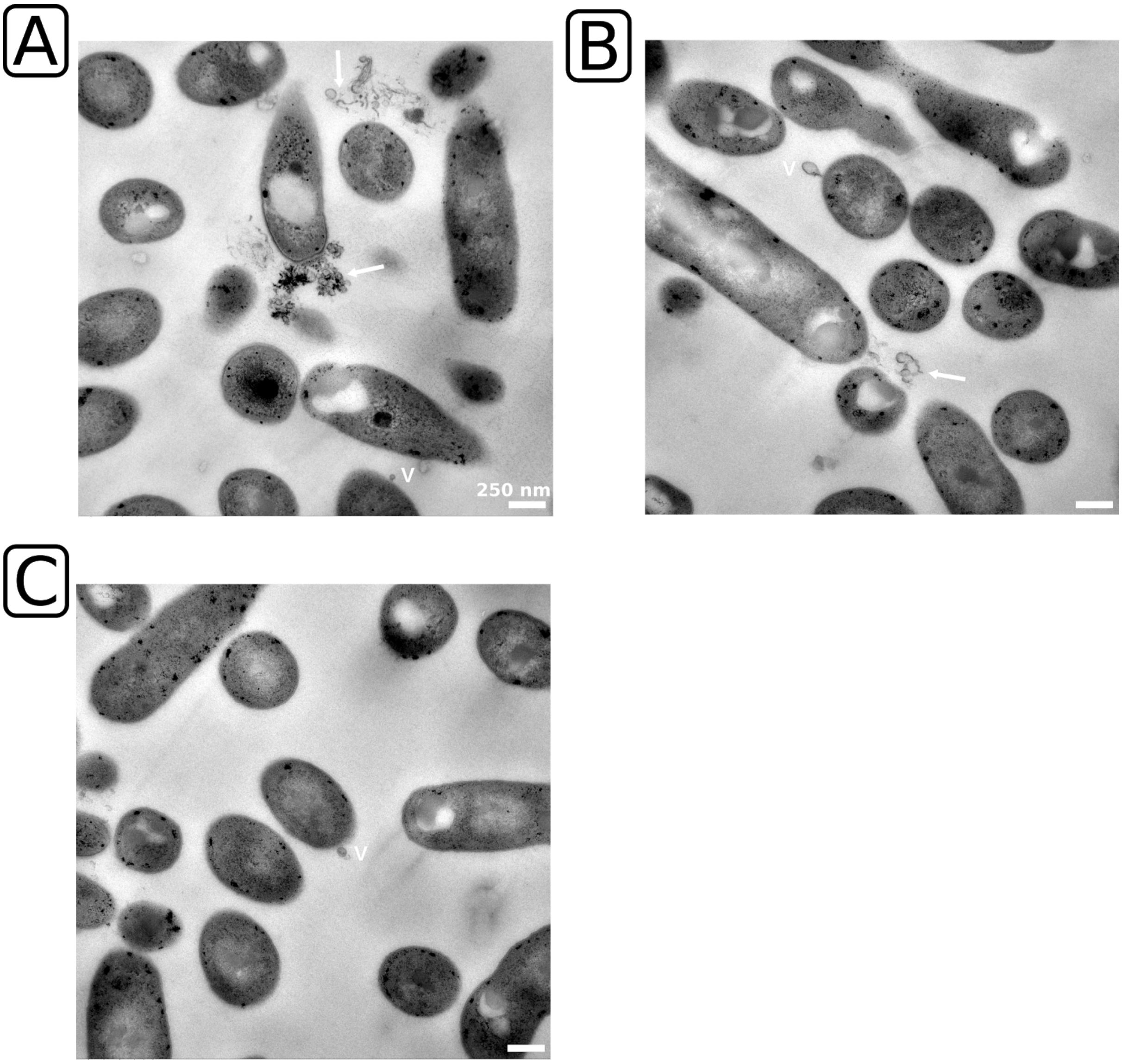

Fig. S2

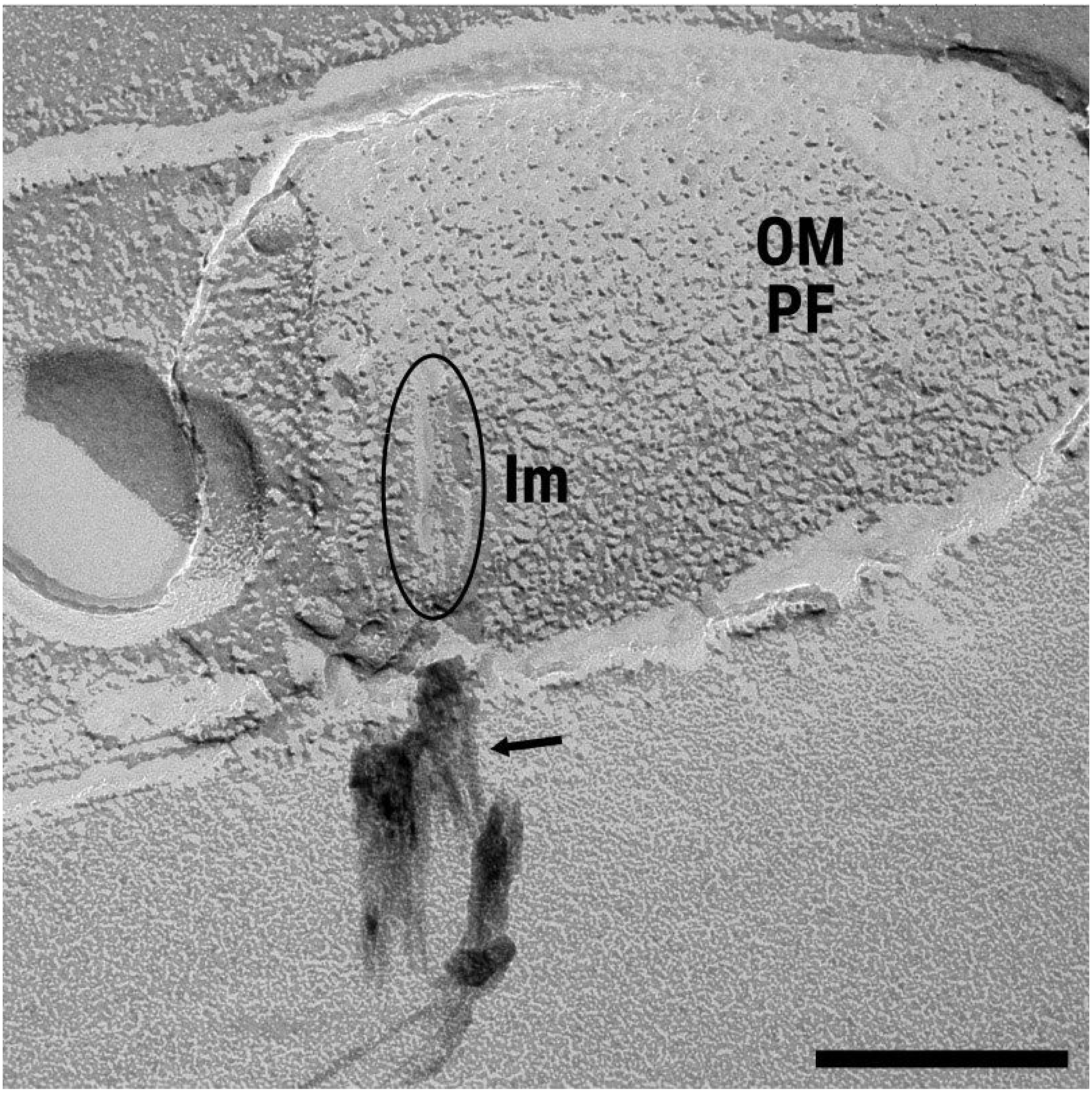

Fig. S3
